# High throughput construction of species characterized bacterial biobank for functional bacteria screening: demonstration with GABA-producing bacteria

**DOI:** 10.3389/fmicb.2025.1545877

**Published:** 2025-03-27

**Authors:** Yanci Qiu, Dingding Fan, Jianxin Wang, Xiaoxue Zhou, Xin Teng, Chitong Rao

**Affiliations:** Bluepha Co., Ltd., Shanghai, China

**Keywords:** Nanopore, barcoding, functional bacteria, biosensor, high-throughput screening, species-characterized bacterial biobank, GABA

## Abstract

Bacteria and their metabolites exhibit remarkable diversity, offering substantial potential for industrial biotechnology. However, the low throughput for constructing and screening bacterial biobanks limits the exploration and utilization of this diversity. In this study, we developed a cost-effective, high-throughput platform for bacterial biobank construction and functional screening. We employed a double-ended barcoding strategy, enabling thousands of bacterial isolates to be pooled for simultaneous Nanopore sequencing of full-length 16S rDNA for species identification. This approach demonstrated 99% accuracy compared to Sanger sequencing while reducing per-sample costs to under 10%. Using this platform, we established a bacterial biobank comprising 15,337 bacterial isolates derived from fermented foods and infant feces collected across China. To identify functional bacteria within the biobank, we designed a versatile fluorescence-based biosensor system employing dual plasmids to decouple metabolite sensing from signal reporting. This modular biosensor framework can be readily adapted for detecting diverse metabolites. As a proof-of-concept, we screened 1,740 isolates and identified 46 with high γ-aminobutyric acid (GABA)-producing capacity, demonstrating potential for probiotic development. Together, our integrated bacterial identification and functional screening platform provides an efficient pipeline for the discovery of functional bacteria, advancing industrial biotechnology through synthetic biology.

## 1 Introduction

Bacteria are ubiquitous on Earth and have a long history of industrial applications, including the production of antibiotics, food products, enzymes, amino acids, vaccines, and fine chemicals (Abdel-Aziz et al., 2017; Sanchez et al., 2018; Wang et al., 2021). Despite significant advancements in molecular biology, synthetic biology, and bioengineering, only a small fraction of bacterial diversity has been characterized and harnessed for commercial purposes (Scott and Piel, 2019). This unexplored diversity holds immense potential for advancing industrial biotechnology.

The identification of novel functional bacteria necessitates their isolation, cultivation, and characterization from environmental sources. Establishing a bacterial biobank comprising diverse, species-characterized strains is a critical foundation for repeated functional screening. However, traditional methods for isolating and identification of individual bacteria from complex microbial ecosystems are labor-intensive and poorly scalable. These limitations cannot be fully addressed through improved culture techniques alone and increasingly rely on high-throughput technologies. Donachie et al. emphasized that understanding the full extent of microbial diversity requires integrated strategies combining high-throughput culturing with molecular biology techniques (Donachie et al., 2007).

Species or genus identification of bacterial isolates in biobanks traditionally depends on 16S rDNA sequencing, which can be performed using various sequencing technologies. First-generation Sanger sequencing provides the highest accuracy for full-length 16S rDNA and remains the gold standard for species identification. However, its relatively high cost per sample hinders its utility in large-scale bacterial biobank projects. Second-generation sequencing (e.g., Illumina) offers lower costs and high throughput by targeting variable regions of the 16S rDNA, such as V3/V4. However, its short read lengths (150–300 bp) may limit resolution and result in inaccurate species-level identification (Johnson et al., 2019). Third-generation sequencing technologies, such as Nanopore and PacBio, enable full-length 16S rDNA sequencing, offering higher precision in species identification. Recent advances in the accuracy of these platforms have demonstrated their potential for species-level identification in microbial community studies (Johnson et al., 2019; Matsuo et al., 2021). For example, Matsuo et al. employed Nanopore sequencing for profiling the human gut microbiota at the species level (Matsuo et al., 2021). Johnson et al. utilized PacBio HiFi (CCS) reads to identify bacterial species and strains by leveraging the multi-copy nature of the 16S rDNA gene (Johnson et al., 2019). Additionally, Srivathsan et al. demonstrated the application of Nanopore-based barcoding for insect diversity analysis (Srivathsan et al., 2021).

Despite these advancements, the application of third-generation sequencing for cost-effective, high-throughput species identification in large-scale bacterial biobanks remains limited. While PacBio HiFi sequencing achieves > 99% accuracy, its sequencing-center operation model and high per-run costs reduce its feasibility for 16S rDNA projects requiring fewer reads. Nanopore sequencing, on the other hand, is limited by error rates ranging from 5 to 20%, depending on molecule type and library preparation (Rang et al., 2018). These errors can impair taxonomic classification accuracy, particularly for closely related species. To apply third-generation sequencing effectively in biobank-scale species identification, technical optimizations are needed in PCR conditions, barcode design for pooled sequencing, and bioinformatics pipelines for demultiplexing and species classification.

Another major challenge lies in functionally screening thousands of microbial isolates from bacterial biobanks, necessitating cost-effective detection methods. Many bacterial metabolites, such as lactate, propionate, and amino acids, are of industrial significance (Abdel-Aziz et al., 2017; Sanchez et al., 2018; Wang et al., 2021), making their rapid detection highly valuable. One such metabolite, γ-aminobutyric acid (GABA), is an inhibitory neurotransmitter with potential health benefits, including mitigating anxiety, stress, and fear. Previous studies have shown that certain bacteria produce GABA as part of their acid resistance mechanisms (Feehily and Karatzas, 2013), including gut bacteria (Otaru et al., 2021) and those in GABA-rich fermented foods (Diez-Gutiérrez et al., 2020), presenting opportunities for developing GABA-producing probiotics. While traditional High-Performance Liquid Chromatography (HPLC) is commonly used for GABA quantification, it is limited to analyzing 50–100 samples per day and incurs high per-sample costs.

Biosensors, a powerful tool in synthetic biology, offer rapid, cost-effective detection of target analytes by converting biological signals into easily interpretable outputs, such as fluorescence. Recent advances in metabolite-based biosensors have enabled high-throughput metabolite analyses, capable of screening over 100,000 samples daily (Li et al., 2022; O’Connor et al., 2024). Leveraging transcription factors (TFs) evolved by nature to respond to a wide range of native metabolites, biosensors represent a promising solution for high-throughput functional screening of bacterial biobanks (Qian and Cirino, 2016; Mannan et al., 2017; Zhou and Zhang, 2023).

In this study, we developed a cost-effective pipeline for large-scale bacterial biobank construction and high-throughput functional screening. The combination of these techniques offers an efficient and streamlined process for unlocking the biotechnological potential of environmental bacterial diversity, bridging fundamental research and practical applications in industrial biotechnology.

## 2 Results

### 2.1 Pipeline for constructing a species-characterized bacterial biobank and subsequent functional screening

**FIGURE 1:**
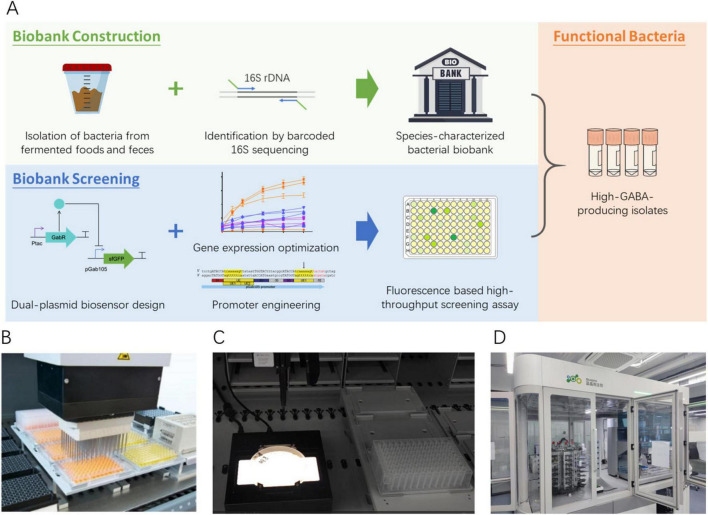
**(A)** Workflow for constructing a species-characterized bacterial biobank and subsequent functional screening. **(B–D)** Customized high-throughput platform.

To harness microbial biodiversity for biotechnological applications, we developed a high-throughput and cost-effective workflow for biobank construction and functional screening (Figure 1A). Single colonies isolated from fermented food and infant feces samples were cultured in 96-well plates containing various growth media as described in Methods. The 16S rDNA regions were amplified using double-ended barcoded primers and a robustly optimized PCR protocol, with the resulting PCR products pooled and sequenced on the Oxford Nanopore PromethION platform. The sequencing reads were analyzed using a customized bioinformatics pipeline to determine the taxonomy of each sample. Once established, the biobank is reusable for screening various functional bacterial traits. For functional screening, we developed a versatile dual-plasmid biosensor system comprising a sensor plasmid and a reporter plasmid. This modular system is highly adaptable, enabling targeted performance optimization through promoter engineering and gene expression adjustments, and it can be tailored to identify diverse functional bacterial phenotypes. In this study, the biosensor was employed to screen the biobank for high-GABA-producing strains. Both the biobank construction and functional screening workflows were implemented on a high-throughput liquid handler platform (Figures 1B–D), which significantly enhanced processing efficiency while reducing labor costs. Notably, a single operator could process up to 2,500 samples per day through PCR amplification and biosensor-based screening.

### 2.2 Cost-effective workflow for large-scale bacterial species identification

**FIGURE 2:**
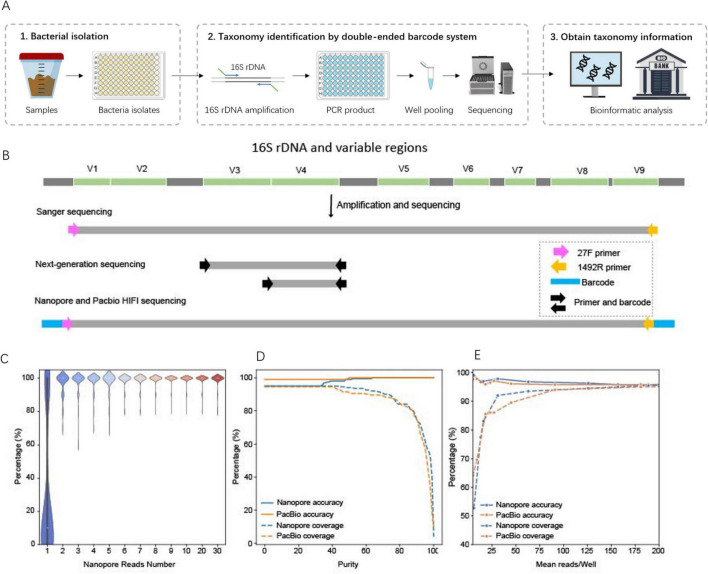
Cost-effective pipeline for large-scale bacterial species identification. **(A)** workflow of large-scale bacterial species identification. **(B)** Primer design for different sequencing platforms. **(C)** Accuracy of species identification using different numbers of Nanopore reads, benchmarked against the sequencing results. For each number of Nanopore reads we sampled 100 times for simulation. **(D)** Effect of the minimal purity threshold on the percentage of samples (coverage) with correct species identification (accuracy). Purity is defined as the relative abundance of the most likely species call (the percentage of reads supporting that species call) in a sample. **(E)** Effect of minimal average of sequencing reads per sample on the percentage of samples (coverage) with correct species identification (accuracy).

To establish a cost-effective pipeline for bacterial species identification based on 16S rDNA sequencing, we designed and optimized a comprehensive workflow, starting with 16S PCR amplification and proceeding through sequencing and species identification (Figure 2A). Using a test set of 340 bacterial isolates derived from infant fecal samples (cultured in four 96-well plates, with one well in the last column of each plate containing *Cupriavidus necator* as a positive control and the others containing culture medium as negative controls), we developed a high-throughput and robust PCR protocol optimized for uniform amplification across diverse bacterial strains. Full-length 16S rDNA amplicons were sequenced using Sanger, Nanopore, and PacBio platforms, while partial 16S rDNA sequencing (V3–V4 and V4 regions) was performed using Illumina 250 bp paired-end sequencing (Rao et al., 2021). A customized bioinformatics pipeline enabled species identification and comparative analysis of sequencing accuracy and cost across platforms.

The 16S PCR protocol was optimized to ensure uniform representation of samples in pooled sequencing, addressing challenges such as varying bacterial growth densities, cell lysis efficiencies, and metabolite compositions. Using a Tecan Freedom EVO^®^ liquid handler, we achieved a streamlined, high-throughput workflow. The optimized one-step PCR method yielded uniform band intensity for 95% of samples in gel electrophoresis, a significant improvement over the initial protocol and two-step PCR method (Supplementary Figure S1). The method was highly cost-effective, with a per-sample cost of $0.21, and robust enough to ensure reliable amplification across a diverse microbial population.

For pooled sequencing of thousands of 16S amplicon samples, we employed double-ended barcodes to the PCR primers (Figure 2B). Specifically, for Nanopore and PacBio sequencing, we designed 75 pairs of 40-bp double-ended barcodes flanking the 16S rDNA primers (27F/1492R), supporting multiplexing of up to 5,625 samples in a single sequencing run. These double-ended barcodes were designed so that they contained no homopolymer sequences, minimizing single-molecule sequencing errors (Jain et al., 2016). Additionally, the barcodes had less than 60% similarity between each other, reducing the likelihood of demultiplexing errors, and no similarity to the official Nanopore barcodes, ensuring compatibility when multiplexing across multiple libraries. When aligned to sequencing reads, there were > 90% similarity between matched barcodes and reads, while only 0.04% of reads were erroneously assigned to multiple barcodes (unexpected hybrid barcodes). Demultiplexing rates were 31.19% for Nanopore and 50.37% for PacBio, with the Nanopore rate falling within the previously reported range of 15.52–43.39% (Srivathsan et al., 2021), both sufficient to recover adequate reads for downstream species identification (Table 1). These results demonstrated that our barcode design and bioinformatics pipeline effectively reduced sequencing errors, enabling precise differentiation of pooled samples.

**TABLE 1:** Demultiplexing summary of Nanopore and PacBio reads.

	Nanopore	Pacbio
Number of samples	340	340
Total reads	14,481,503	1,073,836
Reads with proper length (1,400∼1,800 bp)	11,624,754	1,028,088
Reads with predetermined dual barcodes	40.56%	60.81%
Reads with unexpected hybrid barcodes	0.04%	0.04%
Reads with only one barcode	45.93%	34.24%
Reads without any barcode	13.47%	4.91%
Demultiplexing ratio	31.19%	50.37%

To identify the most suitable sequencing platform for bacterial species identification, we evaluated Sanger, Illumina, Nanopore, and PacBio sequencing technologies, focusing on accuracy and cost per sample. A uniform 16S rDNA database and the *Emu* bioinformatics pipeline (Curry et al., 2022), a tool optimized for error-prone full-length 16S rDNA reads but also applicable to short-read data, were employed for species-level taxonomy identification. This standardized approach enabled a direct comparison of the performance of each sequencing platform for species-level identification of bacterial strains in the biobank.

Sanger sequencing was used as the gold standard to benchmark the results from the other platforms. Among the 340 Sanger sequencing results, 221 bacterial samples with sequence lengths exceeding 1,000 bp and ≥ 99% similarity to the 16S rDNA database were deemed reliable references. For these isolates, species-level identification results from Sanger sequencing were compared to those obtained from Illumina, PacBio, and Nanopore platforms. Discrepancies were observed for 22 isolates, where alignment revealed that Sanger sequences exhibited high similarity (>99%) to multiple species in the database with identical alignment scores. This outcome indicated that 16S sequencing using the 27F/1492R primer pair was insufficient for definitive species-level identification in these cases (Supplementary Figure S2). Consequently, 199 Sanger-derived samples with unambiguous species-level identification were selected as benchmarks for subsequent evaluations.

The Illumina platform, with a single read length of 250 bp, demonstrated 97.76% of bases at Q20 (Phred quality score, indicating 99% base accuracy) (Table 2), comparable to Sanger sequencing. The average sequencing depth per sample ranged from 4,497× to 9,510×. Despite this high sequencing depth, Illumina sequencing of 55 samples, confirmed as single-species based on Sanger, Nanopore, and PacBio data, consistently identified 6–10 species, often including multiple species within the same genus. For instance, *Enterococcus faecium* was detected alongside *Enterococcus faecalis*, *Enterococcus hirae*, and other closely related species. These results align with the known limitations of 16S V3–V4 and V4 regions, which, while adequate for genus-level identification, lack the resolution required for reliable species-level discrimination (Johnson et al., 2019). Based on these observations, Illumina sequencing was excluded from further analysis.

**TABLE 2:** The sequencing summary of four different platforms.

Platform	Sample number	Read length	Total reads number	Q20 bases	Distribution of read number per sample							
					**Min**	**1st quarter**	**Median**	**Mean**	**3rd quarter**	**Max**	**Zero read**	**Coefficient of variation**
Sanger	384	164–1,821 bp	363	97.76%	1	1	1	1	1	1	24	0
NGS V3V4 region	384	250 bp*2	4,441,714	96.64%	7	2,542	4,738	4,497	6,491	10,882	0	0.581
NGS V4 region	384	250 bp*2	9,243,398	96.90%	123	5,471	9,982	9,510	13,973	21,621	0	0.532
Nanopore	384	1,400–1,800 bp	11,624,754	83.25%	5	1,003	5,692	6,296	9,997	30,416	0	0.892
PacBio	384	1,400–1,800 bp	1,028,088	99.13%	1	144	764	907	1,430	5,084	5	0.936

Both Nanopore and PacBio platforms support deep sequencing of full-length 16S rDNA, but Nanopore is associated with lower accuracy. In a typical run, Nanopore yielded 83.25% of bases at Q20, significantly lower than Sanger (97.76%) and PacBio HiFi sequencing (99.13%). To determine the minimum number of reads required for accurate species identification, we simulated bioinformatics processing across 1–30 reads per sample for the 199 benchmark samples (Figure 2C). The results showed that with just two reads, Nanopore achieved an average identification accuracy of 97.3%, which increased to 99.0% with six reads. Based on these findings, we established a threshold of at least five reads per sample to ensure reliable species-level identification with Nanopore sequencing.

To evaluate the practical performance of Nanopore and PacBio in a bacterial biobank context, we conducted high-depth sequencing, achieving an average of 6,296 reads per sample for Nanopore and 907 reads per sample for PacBio (Table 2). Species identification of the 199 benchmark samples revealed that 106 samples (53.26%) using Nanopore and 148 samples (74.37%) using PacBio had two or more bacterial species identified. This impurity likely stemmed from cogrowth of bacterial species, contamination during experimental and sequencing procedures, or the presence of closely related species with highly similar 16S rDNA sequences (see section Discussion). To mitigate this issue, we selected the species with the highest relative abundance as the identified species and defined the relative abundance of this dominant species as purity. A higher purity threshold reduced the proportion of samples successfully identified but increased concordance with the Sanger reference.

Using a 30% purity threshold, 95% of Nanopore samples produced a species call, with 95% of these matching the Sanger reference. For PacBio, these ratios were 99 and 94.5%, respectively (Figure 2D). Simulating species identification with fewer reads (0.5–100% of the actual total reads) showed that sample coverage with species calls ranged from 52.7% to 95.0% for Nanopore and 64.2–95.0% for PacBio (Figure 2E). To achieve > 90% coverage of samples with species calls, Nanopore required a minimal average of 31 reads per sample, while PacBio required 91 reads (Figure 2E). Despite its lower per-read accuracy, Nanopore achieved comparable results to PacBio with fewer reads due to a more uniform distribution of sequencing depth (Table 2).

In conclusion, to achieve greater than 95% accuracy in species identification and over 90% coverage of pooled samples, we established the following thresholds for Nanopore sequencing: a minimum purity of 30%, an average of 31 reads per sample, with at least 5 reads per sample. From an economic perspective, Nanopore emerged as the most cost-effective platform for full-length 16S sequencing. In our test run, Nanopore sequencing cost $1.72 per sample, which is more affordable than Sanger and PacBio sequencing (Table 3). By applying the minimal average sequencing depth threshold for cost optimization, the per-sample cost was further reduced to $0.62 for Nanopore, representing an 80 and 60% reduction compared to Sanger and PacBio, respectively (Table 3). Considering its sufficient accuracy for species identification, significantly lower cost per sample, and the advantages of portability and real-time analysis, Nanopore was selected as the sequencing platform for large-scale bacterial species identification in our workflow.

**TABLE 3:** Cost comparison of different platforms.

Cost breakdown		Sanger	Nanopore	PacBio
PCR cost per sample ($)		0.21	0.21	0.21
Cost per library construction ($)		0.71*2	155.59	282.89
Actual scenario	Pooled samples	1	384	384
	Average sequencing depth per sample	1	6,296	907
	Cost per library sequencing ($)	0.71*2	424.33	2,263.08
	Total cost per sample ($)	3.04	1.72	6.84
Optimal scenario	Pooled samples	1	5,625	5,625
	Minimal average sequencing depth per sample	1	31	91
	Cost of library sequencing ($)	0.71*2	34.79	3,315.06
	Total cost per sample ($)	3.04	0.24	0.85

### 2.3 Construction of a species-characterized bacterial biobank with wide geographical coverage

**FIGURE 3:**
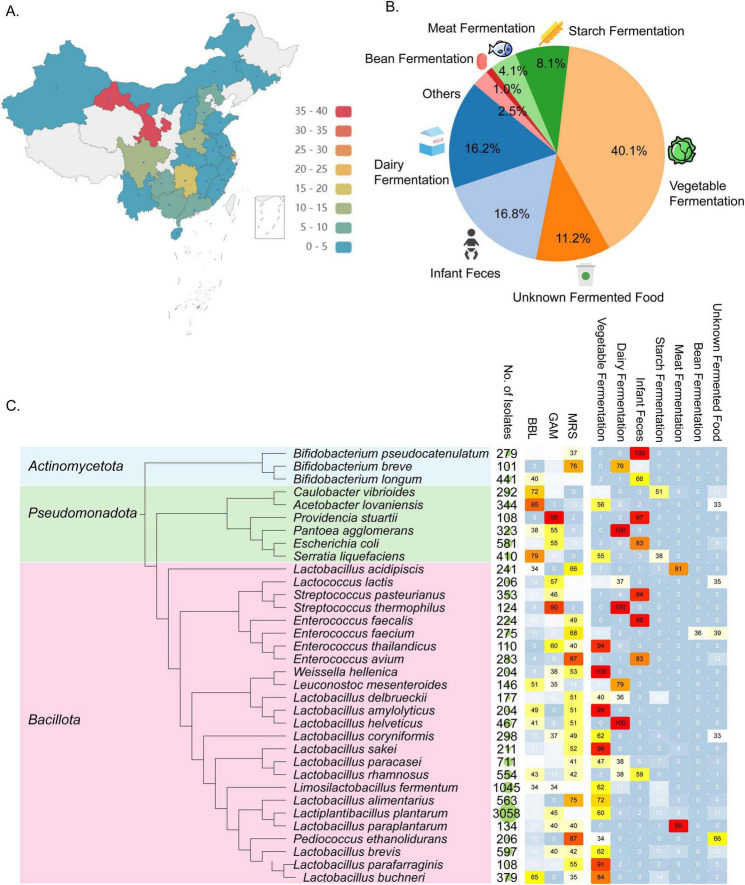
Overview of bacterial strains identified through large-scale Nanopore sequencing. **(A)** Geographical locations where samples were collected across China. **(B)** Samples categorized based on their origins. **(C)** Phylogenetic tree of bacterial species that were identified from > 100 isolated samples. Accompanying the tree are the number of samples covering each species, the percentage of culture media used to isolate each species, and the distribution of sample categories from which each species was recovered.

To establish a species-characterized bacterial biobank for identifying GABA-producing and other potentially functional bacteria, we collected 197 fermented foods and healthy infant feces samples, representing a wide geographical range across 22 provinces, municipalities, and autonomous regions in China (Figure 3A). The largest proportion of samples came from vegetable-fermented foods (40.1%), followed by infant feces (16.8%), dairy products (16.2%), and starch fermentation (8.1%) (Figure 3B). Bacterial isolation was performed using a variety of culture media using a custom designed liquid handling system (see section Methods), and a total of 21,120 bacterial isolates were sequenced in 10 batches, each containing 24 96-well plates, using the Nanopore platform. Consistent with the test run, the barcode demultiplexing rates ranged from 28 to 36% across different batches, demonstrating the high reproducibility of our double-ended barcoding strategy (Supplementary Table S1). By applying a purity threshold of greater than 30% and a minimum of 5 reads per sample, we successfully identified species for 15,337 isolates, encompassing three distinct phyla and 209 species. This diversity is particularly valuable for applications in the food industry (Figure 3C).

### 2.4 Design and optimization of dual-plasmid GABA biosensor

A dual-plasmid biosensor system was developed using GABA as an exemplar for high-throughput screening of functional bacteria from an established bacterial biobank. This biosensor, based on a prototype GABA sensor, utilizes the GabR transcriptional repressor from *Bacillus subtilis* (Lebovich and Andrews, 2022). In the presence of GABA, this repression is alleviated, leading to the activation of downstream sfGFP expression. The GabR sensor and the sfGFP reporter were modularly positioned on separate plasmids, allowing for flexible use and easy modification to detect other metabolites.

**FIGURE 4:**
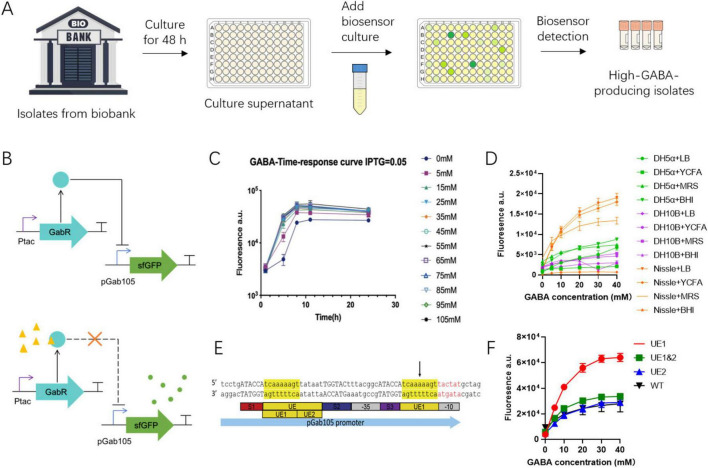
Optimization of dual-plasmid GABA biosensor. **(A)** Workflow of using GABA biosensor to screen for high-GABA-producing bacteria from an established bacterial biobank. **(B)** Design of the dual-plasmid system biosensor. When GABA is absent, the IPTG-induced GabR transcriptional repressor represses the pGab105 promoter, and the expression of the downstream sfGFP. When GABA is present (yellow triangle), GABA binds to GabR so that the transcriptional repressor and loses its repressive effect on pGab105, and sfGFP is expressed (green circle). **(C)** Time response curve of the 6-h GABA biosensor. **(D)** The performance of the pGab105 promoter in different *E. coli* strains and different culture media. **(E)** Structure of the improved pGab105 promoter, where UE1 is inserted upstream of the - 10 region of the original pGab105. **(F)** The fluorescence readouts of different biosensor systems in response to different GABA concentrations.

The dual-plasmid biosensor was introduced into *E. coli* for screening GABA-producing bacteria. Initially, the GABA biosensor was prepared by culturing in LB medium for 6 h, with IPTG added to induce the sensor expression, followed by inoculation with the culture supernatant of bacterial isolates from the biobank and further cultivation at 37°C (Figure 4A). GABA concentrations were then determined by measuring fluorescence in a microplate reader (Figure 4B). It was found that endogenous GabR expression in *E. coli* had a negligible effect on the measured GABA concentration, although a relatively high fluorescence background was observed (Supplementary Figure S3).

The performance of the GABA biosensor was then optimized through promoter engineering, chassis optimization, and culture condition optimization. The sensor’s dynamic range was first optimized by varying IPTG concentrations, revealing comparable performance across different IPTG concentrations (Supplementary Figure S4). A time response curve was then tested over 24 h to determine the optimal culture time for the biosensor’s ability to distinguish different GABA concentrations. The results showed that the response signal saturated at GABA concentrations above 20 mM, with the fluorescent signal plateauing at 8 h (Figure 4C).

To mimic actual biosensor usage conditions, a non-GABA-producing *Lactiplantibacillus plantarum* strain was cultured in various media. The culture supernatants were then used to culture the GABA biosensor in different media and chassis. *E. coli* Nissle 1917, cultured in BHI or LB, exhibited the highest maximum activation readout (Figure 4D). Additionally, the pGab105 promoter sequence was optimized by relocating an AT-rich sequence (UE1) within the GabR binding site (UE) closer to the −10 region of the pGab105 promoter (Figure 4E). This modification significantly enhanced the dynamic range and reduced background activation (Figure 4F). With the modified sensor in *E. coli* Nissle 1917, the background was reduced by 60%, and the maximum activation readout increased to 220% of the original biosensor.

To assess the impact of complex constituents in culture supernatants on GABA biosensor detection, culture supernatants from various bacteria (*Lactiplantibacillus plantarum*, *Lactobacillus reuteri*, *Enterococcus avium*, *Escherichia coli*, and *Bifidobacterium animalis*) were tested. Initial GABA concentrations were measured, and known amounts of 2–10 mM GABA were spiked into the samples. Detection was performed using the GABA biosensor and ultra-performance liquid chromatography-high-resolution mass spectrometry (UPLC-HRMS). The results from the optimized GABA biosensor showed a high correlation with those from UPLC-HRMS (*p* < 0.0001), validating the biosensor’s accuracy for real screening scenarios (Supplementary Figure S5).

### 2.5 Identification of high-GABA-producing bacteria from the established bacterial biobank

**FIGURE 5:**
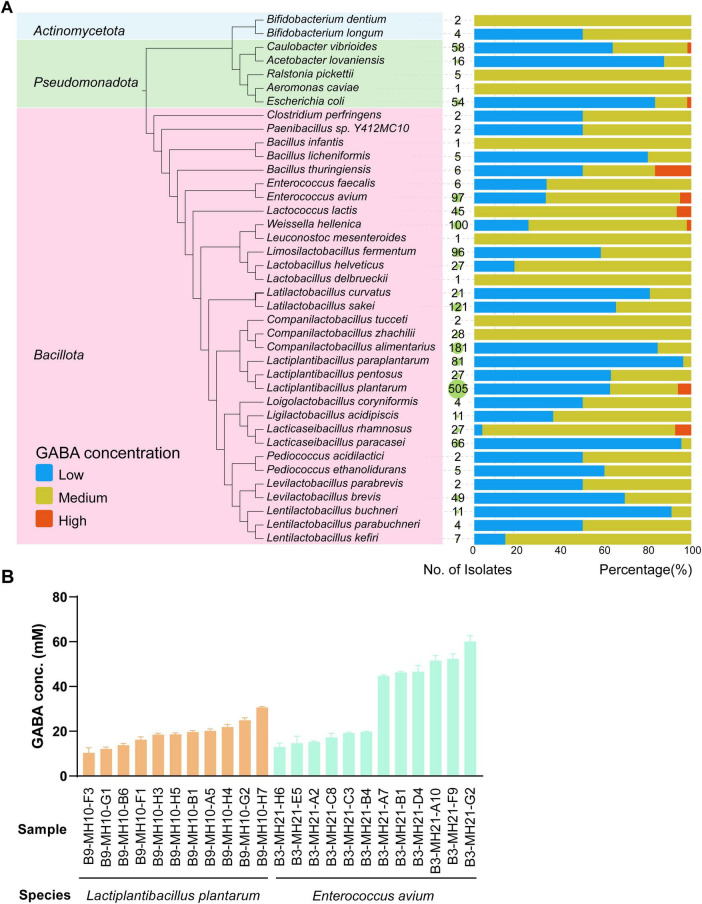
High-throughput screening for GABA producing bacteria. **(A)** Overview of GABA production levels of 1,740 bacterial samples screened using the optimized biosensor. The number and green circles in the middle represent the total number of bacterial isolates of the corresponding species tested; the stacked bar graph on the right represents the percentage of isolate with low, medium or high GABA production. **(B)** GABA production analyzed by UPLC-HRMS of selected high-GABA producing bacteria obtained from the biosensor screening.

We then applied the optimized GABA biosensor to screen for high-GABA-producing bacteria from the established bacterial biobank. In the screening process, we categorized bacterial isolates based on their GABA production levels as detected by the biosensor. Isolates producing less than 5 mM GABA (with fluorescence readout below 20,000 AU) were classified as low- or non-GABA-producing bacteria; those producing 5–20 mM GABA (fluorescence readout between 20,000 and 60,000 AU) as medium-GABA-producing bacteria; and those producing more than 20 mM GABA (fluorescence readout exceeding 60,000 AU) as high-GABA-producing bacteria (Supplementary Figure S6). Out of 1,740 bacterial isolates screened from the biobank, 628 isolates (36%) were identified as medium-GABA producers, while 46 isolates (2.6%) were classified as high-GABA producers (Figure 5A). These high-GABA-producing isolates were derived from seven different fermented food or infant feces samples. To validate the biosensor results, 23 high-GABA-producing isolates originating from two samples were randomly selected for further analysis using UPLC-HRMS. Representative MS EIC of GABA standard and bacterial sample are shown in Supplementary Figure S7. This analysis confirmed that these strains produced GABA at concentrations ranging from 10 to 50 mM (Figure 5B).

## 3 Discussion

The exploration of the microbial world has long been a focal point of scientific research. Microorganisms, particularly bacteria, play essential roles in various ecological processes and hold significant biotechnological potential. However, accessing and identifying functional bacteria from complex samples remains a challenging task. To address this, it is crucial to develop high-throughput methods for the identification of functional bacteria. This includes the establishment of a species-characterized bacterial biobank and the implementation of efficient screening methods to identify functional bacteria within these bacterial biobanks. Such advancements will significantly enhance our ability to harness the potential of bacteria for ecological and biotechnological applications. To overcome these challenges, the construction of a species-characterized, comprehensive bacterial biobank would offer a valuable resource, preserving the genetic and phenotypic diversity present in natural microbial communities. However, establishing such a biobank can be time-consuming and costly, particularly when dealing with large numbers of bacterial isolates. Our high-throughput approach enables the efficient capture and representation of a broad range of bacterial species and strains from diverse sources, providing a rapid and effective tool for individual research groups to access comprehensive culture collections from ecological samples.

To contextualize our approach within the current state of the field, we compared our barcoding strategy with established sequencing methodologies, including short-read next-generation sequencing (NGS) platforms, long-read sequencing approaches, and existing commercial barcoding kits. While several barcoding strategies have been used for 16S rRNA sequencing, most custom approaches have been optimized for short-read sequencing platforms like Illumina, which employ dual-indexing for error correction and high-throughput multiplexing (Johnson et al., 2019). However, due to their short read lengths (<300 bp), these methods rely on short barcodes (6–13 nt) and can only sequence partial 16S rRNA genes, limiting species-level resolution (Abellan-Schneyder et al., 2021; Pjevac et al., 2021). Sanger sequencing, as the gold standard for full-length 16S rDNA sequencing, is impractical for high-throughput applications due to its high cost. Long-read sequencing platforms like Nanopore and PacBio HiFi (CCS) enable full-length 16S sequencing, but existing methods primarily rely on commercial kits (Matsuo et al., 2021) that use identical 24-bp barcodes, restricting sample multiplexing to 96 samples per sequencing run, leading to higher sequencing costs.

To overcome these limitations, we developed a robust one-step PCR approach, which is more efficient and cost-effective than conventional two-step methods. Additionally, we designed distinct 75 × 75-bp dual-barcodes for each end of the full-length 16S rRNA (∼1,600 bp), significantly improving demultiplexing performance while maintaining high sequencing throughput. Compared to ONTbarcoder (Srivathsan et al., 2021), which employs 13-bp barcodes for COI sequencing (∼700 bp) and achieves demultiplexing ratios of 15.5–48.97%, our strategy demonstrated greater stability (28–36%), making it more reliable for large-scale bacterial species identification. Unlike commercial kits, which allow only 96 samples per sequencing run, our approach significantly increases multiplexing capacity and reduces sequencing costs, making it a scalable and cost-efficient alternative.

In the process of developing analytical methods for species identification, we encountered a significant proportion of samples with less than 100% taxonomic purity. Bacteria isolated from natural samples often associate with other bacteria (Zhang et al., 2021; Huang et al., 2023). For example, Borges et al. found that 88% of isolates from fecal samples were pure (Borges et al., 2023), while Zhang et al. reported that only 45% of bacteria cultivated from plant roots were pure (Zhang et al., 2021). In addition to co-growth of bacterial species, other contributing factors include contamination during experimental procedures, such as aerosols, reagents, and equipment, as well as sequencing errors. In fact, Zhang et al. found that bacteria with a purity greater than 95% were likely to be pure when re-streaked on agar plates, consistent with the assumption that low-abundance sequences might stem from multiple polymorphic copies of the 16S rDNA gene or sequencing errors (Zhang et al., 2021). In our practice, 41% of the 15,337 isolates identified at the species level from fermented foods and infant feces had a purity of more than 95%. Further purification of bacterial strains of interest may be required to generate high-quality stocks for downstream applications.

Once a bacterial biobank is established, high-throughput screening methods enable rapid and efficient identification of functional bacteria, such as those producing valuable metabolites like lactate, propionate, and amino acids. Many organisms have evolved transcription factors that respond to native metabolites, and advances in synthetic biology have made these metabolite-responsive transcription factors versatile for high-throughput screening (Qian and Cirino, 2016; Mannan et al., 2017; Zhou and Zhang, 2023). Using well-insulated genetic designs and characterized promoter parts, we designed a dual-plasmid biosensor system with inducible expression of the metabolite-responsive regulator. The dual-plasmid biosensor system allows separate regulation of the transcriptional factor and its binding site, making the modular biosensor system flexible for various experimental uses and different metabolites. We demonstrated that this biosensor could be a high-throughput approach for identifying functional bacteria from a constructed bacterial biobank, using GABA as an example. Furthermore, the flexibility of the biosensor system allows for easy modification to detect other metabolites of interest. To adapt the system, the transcriptional repressor in the sensor plasmid can be replaced with a repressor that responds to the metabolite of interest, while the promoter in the reporter plasmid should be replaced with the corresponding promoter that is activated by the metabolite.

Our biosensor system distinguishes itself from conventional microbial biosensors by offering enhanced sensitivity and a broader dynamic range. A key application of our biosensor is GABA production detection, which serves as a representative case study demonstrating its effectiveness. We optimized the sensor component, significantly improving its performance over previous versions. Traditional detection methods, such as high-performance liquid chromatography (HPLC), are widely used for metabolite quantification but are inherently low-throughput, processing only three samples per hour (Le et al., 2020). Our biosensor, by contrast, achieves an over 150-fold increase in throughput, allowing for the analysis of more than 500 samples per hour. Additionally, HPLC requires $5.5 per sample, whereas our biosensor achieves comparable accuracy at a cost of only $0.16 per sample, representing a substantial reduction in cost. These advantages make our biosensor an efficient and scalable alternative for metabolite detection, particularly in high-throughput screening applications where speed and cost are critical factors.

In addition to the high-throughput screening process, we developed a flexible automated platform for liquid handling, which enables the processing of up to 5,000 samples per day. This significant throughput allows for efficient and rapid identification of functional bacteria, drastically reducing the need for manual labor and enhancing workflow scalability. However, it is important to note that while this automation brings cost savings in terms of personnel and increases the speed of the workflow, it requires considerable infrastructure, including liquid handling robots and specialized systems for analysis. The infrastructure used for this workflow was not established specifically for this project but can be reused for other technology projects. The investment in these technologies, while reducing operational time and errors, represents a barrier for laboratories with limited access to such equipment. Nevertheless, the workflow we developed can still be executed without this infrastructure. It can be accomplished through manual work or with small-scale automation solutions, making it adaptable for research settings with different resource levels. Future efforts may focus on reducing costs or optimizing workflows to increase accessibility and reproducibility across a broader range of research environments.

GABA is thought to play a major role in controlling nerve cell hyperactivity associated with anxiety, stress and fear, thus suggesting its role in modulating mental health. Previous studies have shown that GABA can be produced by different kinds of bacteria (Smith et al., 1992; Cotter et al., 2001; Pokusaeva et al., 2017; Cui et al., 2020; Otaru et al., 2021), acting as a protective mechanism in bacteria against acid stress (Feehily and Karatzas, 2013; Otaru et al., 2021). We screened 1,740 bacterial isolates from the bacterial biobank we established for their GABA production, and found 38.6% of isolates produces more than 5 mM GABA in 48-h cultivation, consistent with the notion that GABA production is commonly found in bacteria in fermented foods and human feces. In addition, 46 high-GABA-producing strains were identified, holding the potential of being used as GABA producing probiotics for attenuating anxiety and insomnia (Yu et al., 2020).

The GABA production of 23 purified isolates originating from 2 samples was confirmed by UPLC-HRMS. GABA production analyzed by UPLC-HRMS was slightly different from that detected by GABA biosensor, as samples were collected from two different sets of experiments and analyzed by different methods. Among the 23 isolates, 11 *Lactiplantibacillus plantarum* isolates were from Jishou sour meat, and 12 *Enterococcus avium* isolates from one infant fecal sample. Interestingly, *Lactiplantibacillus plantarum* isolates all showed similar GABA production, while *Enterococcus avium* isolate exhibited two distinct levels of GABA production (Figure 4C). This discrepancy is likely due to the simpler environment of the fermented food, which was dominated by a few strains, with the *Lactiplantibacillus plantarum* isolates being the same strain (Marco et al., 2017; Voidarou et al., 2020). In contrast, the complex fecal community might contain at least two different *Enterococcus avium* strains.

## 4 Conclusion

In this study, we developed a cost-effective method for bacterial biobank construction and functional bacteria screening. A species characterized bacterial biobank of 15,337 bacterial isolates was established and 46 high-GABA-producing isolates were identified. This study provides practical tools and valuable resources for leveraging bacteria in industrial biotechnology applications. Future research can build upon these methods to expand the characterization of bacterial diversity and explore additional applications of functional bacteria in various biotechnological fields.

## 5 Experimental procedures

### 5.1 Sample sources and bacterial isolation

Various traditional fermented foods were collected across China, through either donations or purchases. Samples were kept in their designated storage conditions prior to subsequent processing. Fecal samples were collected from 6-months to 3-year-old infant/children and immediately stored at −80°C in 25% glycerol supplemented with 0.5 g/L L-cysteine HCl prior to subsequent processing.

#### 5.1.1 Isolation of bacteria from fecal samples

Single colonies were isolated by the pour plate method. Fecal samples were transferred into anaerobic chamber (Defendor AMW500) with an atmosphere consisting 20% hydrogen and 80% nitrogen at 37°. Inside the anaerobic chamber, the fecal samples were thawed, resuspended, diluted and cultivated on MRS agar plates (Huankai Microbial Sci.&Tech. Co., Ltd., Guangdong, China), BBL agar plates (Hope Bio-Technology Co., Ltd., Qingdao, China) and GAM agar plates (Hope Bio-Technology Co., Ltd., Qingdao, China), and then incubated for 48 h. After incubation, single colonies were picked into 96-well plates and propagated on the corresponding culture media for another 48 h in anaerobic chamber.

#### 5.1.2 Isolation of bacteria from fermented foods

Single colonies were isolated by the pour plate method. Fermented food samples were resuspended, diluted and poured on MRS agar plates (Huankai Microbial Sci.&Tech. Co., Ltd., Guangdong, China), BBL agar plates (Hope Bio-Technology Co., Ltd., Qingdao, China) and GAM agar plates (Hope Bio-Technology Co., Ltd., Qingdao, China) under normal atmospheric condition, and then incubated in sealed boxes with AnaeroPouch (Mitsubishi, Japan) at 37°C for 48 h. Single colonies were then isolated using a custom automated imaging and colony-picking system. Briefly, plates were first placed on an illuminated platform where they were imaged, then single colonies were identified by in-house software and picked into 96-well plates with corresponding culture media using a robotic arm. The 96-well plates were then incubated with AnaeroPouch (Mitsubishi, Japan) at 37°C for another 48 h.

### 5.2 Barcoded primers design

The barcodes for Nanopore and Pacbio are generated using barcode_gen_nanopore.^1^ They are 40 base pairs (bp) in length, with a Levenshtein distance of 16 bp between each other. The GC content is set between 0.4 and 0.6, with no homopolymers. To validate the barcode sequences, we performed self-alignment using blastn, and filtered out candidate barcodes with the following criteria: (1) self-complementary sequences where the complementary length is greater than 6 bp; (2) a similarity greater than 60% and an alignment length greater than 20 bp between each other; (3) barcode sequence aligned with the 16S rDNA database^2^ (4) with an alignment to 16S rDNA similarity greater than 90% and an alignment length greater than 20 bp. Then, seqfold^3^ was used to calculate the thermodynamic free energy of the barcode sequences with primers added (total ∼60 bp), and the barcodes with a free energy greater than −5 were retained. Eventually, 75 pairs of barcoded primers were obtained for amplifying the full-length 16S rDNA sequence. For Illumina sequencing, the barcoded primers to amplify the V3V4 and V4 regions of 16S rDNA are from existing literature (Rao et al., 2021).

### 5.3 Construction of 16S amplicon libraries

The amplicons were prepared in 96-well plates with the Tecan Freedom EVO^®^ platform. For each bacterial sample, 50 μL culture was used and centrifuged at 4,000 g for 99 s to obtain bacterial pellets. After washing the bacterial cells once with 40 μL pure water, 40 μL ALP lysis buffer (0.15 g/mL PEG200, 46.5 mM KOH, pH 13.4) was added, mixed by pipetting and heated at 98° for 15 min. The lysis supernatant was taken after centrifugation at 4,000 g for 99 s for amplification in the following 40 μL PCR reaction:

**Table:** 

Component	Volume (μL)	Note
Template	1	Lysis supernatant
Forward primer	1.5	AGAGTTTGATC**M**TGGCTCAG with barcode
Reverse primer	1.5	GGYTACCTTGTTA**Y**GACTT with barcode
Water	16	
Taq DNA Polymerase	20	BestEnzymes Biotech Co., Ltd. Ref: EG15139-M/L

The PCR reactions were performed using the following program:

1. 94°C for 5 min
2. 94°C for 30 s
3. 55°C for 30 s
4. 72°C for 1 min 30 s

Repeat for 35 cycles

5. 72°C for 10 min.

### 5.4 16S rDNA sequencing and reads preprocessing

The PCR amplification products were pooled in equal volume and sequenced in the Bena sequence center (Henan, China). These amplicon libraries were constructed using standard methods of the corresponding platforms and sequenced as normal together with other clients’ libraries. For Nanopore, the PromethION sequencer was used; for PacBio, the PacBio Sequel II HiFi sequencing was used; for NGS, the Illumina NovaSeq was used under the pair-end 250 bp mode; for Sanger sequencing, the 3,500 Dx was used.

The raw data obtained from Nanopore sequencing were in the fast5 format. Base calling and barcode recognition were performed using GUPPY v5.0.16.^4^ After the format conversion to fastq, the adapter sequences were removed using Porechop v0.2.3. Sequences with a quality score less than 10 were filtered out using NanoFilt v2.7.1. Finally, we used cutadapt v3.5 to recognize and remove primer sequences and perform length filtering to obtain clean 16S sequences. The data obtained from PacBbio sequencing were processed using pa-ccs v6.3.0^5^ to obtain fastq reads. For both Nanopore and Pacbio reads, length filtering is performed to keep clean 16S sequences between 1,400 and 1,800 bp. See Data Availability section for depository details of the sequencing data.

### 5.5 Sample demultiplexing and 16S-based taxonomic identification

To demultiplex Nanopore and PacBio barcoded reads, fasta sequences were converted from fastq reads using seqkit and were aligned against the predetermined barcode sequences using blastn following the criteria: (1) the similarity greater than 90%; (2) the alignment length greater than 35 bp; (3) the sequence length between 1,400 and 1,800. Demultiplexing was carried out based on the alignment using custom scripts (see Data Availability for the scripts depository). For Illumina sequencing, paired-end reads were demultiplexed using split_libraries.py, a QIIME-dependent script, and a mapping file linking barcodes to samples. The resulting forward and reverse fastq reads were split into samples using a QIIME-dependent script split_sequence_file_on_sample_ids.py, and primer sequences were removed using TagCleaner v0.16. These demultiplexed Nanopore, PacBio, and NGS clean reads were subjected to the *Emu* pipeline^6^ for taxonomic identification and abundance profiling. For Sanger sequencing, the full-length 16S rDNA sequence was aligned against the 16S rDNA database of *Emu*, and the hit with the highest alignment score was taken as the species identification result.

To determine the optimal number of Nanopore or PacBio reads for accurate species identification, we randomly selected 199 bacterial samples each with more than 100 clean reads, and then randomly sampled between 1 and 30 reads and ran 100 iterations using the *Emu* pipeline. The proportion of species identifications consistent with the Sanger sequencing results was used as the accuracy at specific sequencing depth.

To evaluate whether reducing the average number of reads per sample could still maintain reliable species identification, we simulated the impact of varying read counts on the accuracy of species identification and the coverage of samples with a confident species call. We randomly sampled from 0.5 to 100% of the demultiplexed data and subjected these subsamples to the *Emu* pipeline for species identification. For each subsample, we calculated the accuracy (defined as the proportion of species identifications consistent with Sanger results) and the sample coverage (defined as the proportion of samples with an accurate species call).

### 5.6 Development of dual-plasmid GABA biosensor

Report plasmid (Supplementary Figure S8) was developed and modified from the standardized BioBrick vector pSB4C5. Sensor plasmid (Supplementary Figure S9) was developed and modified from the traditional p15A-AmpR plasmid. After the pGab105-sfGFP and pGabR DNA sequences were synthetized, they were inserted into the corresponding vectors using the Golden Gate assembly method (see Data Availability for the plasmid map depository). The constructed plasmids were then co-transformed into specific *E. coli* strains and cultured in LB medium with 100 μg/mL ampicillin and 23 μg/mL chloramphenicol. All reagents used for molecular cloning were purchased from Sangon Biotech (Shanghai, China).

The genetically encoded sensor and report plasmids were transformed into *E. coli* and streaked onto LB plate with 50 μg/mL ampicillin and 24 μg/mL chloramphenicol and incubated at 37°C overnight. A single colony was picked and inoculated into liquid LB and cultured at 37°C, 220 rpm overnight. Then 1 mL of the bacteria suspension was inoculated it in a 100 mL LB medium with 50 μg/mL ampicillin and 24 μg/mL chloramphenicol and cultured in a shake flask at 37°C, 220 rpm for 6 h. After incubation, the sensor cell was inoculated into test medium with different pre-set conditions, with 1% inoculation ratio (v/v) and incubated in shaking incubator at 37°C/220 rpm. After incubation in corresponding condition, 15μL sample was taken and added into 135 μL PBS (2g/L-Kan) to prepare for flow cytometry analysis.

### 5.7 High-throughput screening for GABA-producing bacteria

#### 5.7.1 Biosensor strain preparation

GABA biosensor strain was grown on LB plate with 50 μg/mL ampicillin and 24 μg/mL chloramphenicol at 37°C overnight. A two-step activation procedure was used to minimize fluorescent background. Specifically, a single colony was inoculated into liquid LB medium and grown at 37°C and 220 rpm for 6 h. Then 1 mL of the bacteria culture was re-inoculated into 100 mL fresh LB medium with 50 μg/mL ampicillin and 24 μg/mL chloramphenicol and cultured in a shake flask at 37°C and 220 rpm overnight before being mixed with samples to be detected.

#### 5.7.2 GABA detection of bacterial samples using the biosensor

Bacterial isolates in the 96-well format bacterial biobank were cultured for 48 h in the conditions corresponding to their isolation. After centrifugation at 5,000 g for 5 min, 600 μL of the culture supernatant was taken and mixed with 30 μL GABA biosensor strain prepared as above in 96-well plates. The plates were sealed with a breathable sealing film (Corning, BF-400-S), cultured at 37°C, 800 rpm for 6 h and ready for fluorescence detection.

#### 5.7.3 Flow cytometry detection

Fluorescence was measured using the Agilent NovoCyte 2100YB cytometer using the FITC channel (488 nm excitation, bandpass filter with a detection range of 525 ± 20 nm) along with the forward-scatter and side-scatter for noise filtering. For each sample, a total of 20,000 gated cell events were collected and the geometric median fluorescence was calculated in Flow Jo v10.8.1.

#### 5.8 Mass spectrometry detection of GABA production

#### 5.8.1 Standard curve preparation

A 3 mg/L GABA standard stock with 0.5 mg/L 4-Aminobutyric acid-2,2,3,3,4,4-d6 (GABA_d6, Sigma Aldrich) internal standard was first prepared in water. The stock was then diluted to 0.03, 0.09, 0.15, 0.3, 0.45, 0.75, 1.05 mg/L GABA with 0.5 mg/L GABA_d6 internal standard aqueous solution.

#### 5.8.2 Sample preparation

A 80 μL of the sample was added to 1,000 μL of pre-chilled 80% methanol aqueous solution (stored at −20°C). The mixture was vortexed and incubated on ice for 5 min to allow protein precipitation. Afterward, the samples were centrifuged at 15,000 g at 4°C for 30 minutes, and the supernatant, which contains the GABA extract, was collected in HPLC vials (Agilent, United States) for further analysis.

#### 5.8.3 UPLC—HRMS analysis

UPLC—HRMS analysis: GABA concentrations of extracts were analyzed using an UltiMate 3000 Basic HPLC Systems coupled to a Q Exactive HF-X mass spectrometer (Thermo Fisher). Chromatographic separation was performed using an ACQUITY UPLC BEH C18 reversed phase C18 column 2.1 × 100 mm with 1.7 μm particle size (Waters). Column temperature was set to 45°C, the sample temperature was set to 4°C and the injection volume was 2 μL. Eluent A consisted of MQ water with 0.1% v/v formic acid, and eluent B consisted of methanol with 0.1% v/v formic acid. The following gradient was applied: 0 min, 2% B; 1 min, 2% B; 2.5 min, 98% B; 4.5 min, 98% B; 4.6 min, 2% B; 6.0 min, 2% B, and the eluent flowed at a rate of 0.15 mL/min. The total runtime is 6 min.

Electrospray ionization in positive mode was used for GABA. The mass spectrometry was run in selective reaction mode (SRM). For MS acquisition, the precursor/product ion pairs for GABA were m/z 104.07 → m/z 87.04. For MS/MS acquisition, the parameters include a resolution of 30,000, an AGC target of 2.00 × 105, and a maximum ion injection time (IT) of 100 ms. The isolation window is set to 0.5 m/z. Collision energy (CE) is applied at 10 eV for fragmentation. Data acquisition and quantification were performed using Xcalibur. Quantification was performed against a linear regression curve based on the duplicate injection of calibration standards.

## Data Availability

The datasets presented in this study can be found in online repositories. The names of the repository/repositories and accession number(s) can be found at: https://www.ncbi.nlm.nih.gov/, PRJNA1170062.

## Footnotes

1
https://github.com/Sn0flingan/barcode_gen_nanopore

2
https://github.com/treangenlab/emu

3
https://github.com/Lattice-Automation/seqfold

4
https://timkahlke.github.io/LongRead_tutorials/BS_G.html

5
https://ccs.how/

6
https://github.com/treangenlab/emu
