# Supplementary material for: High throughput construction of species characterized bacterial biobank for functional bacteria screening: demonstration with GABA-producing bacteria
Source: Front Microbiol. 2025 Mar 27;16:1545877. doi: 10.3389/fmicb.2025.1545877 (PMC11984947; doi:10.3389/fmicb.2025.1545877)
Supplement: Supplementary file 1 [file Data_Sheet_1.docx]

Supplementary Material

**High throughput construction of species characterized biobank for functional bacteria screening: Demonstration with GABA-producing bacteria**

Yanci Qiu^1§^, Dingding Fan^1§^, Jianxin Wang^1^, Xiaoxue Zhou^1^, Xin Teng^1^*, Chitong Rao^1^*

^1^ Bluepha Co. Ltd., Shanghai, China

^§^ Theses authors contributed equally to this work.

*Correspondence

Chitong Rao, [raochitong@gmail.com](mailto:raochitong@gmail.com)

Xin Teng, [tengxin@bluepha.com](mailto:tengxin@bluepha.com)

No. 210, Lane 345, Guangyue Road, Hongkou District, Shanghai


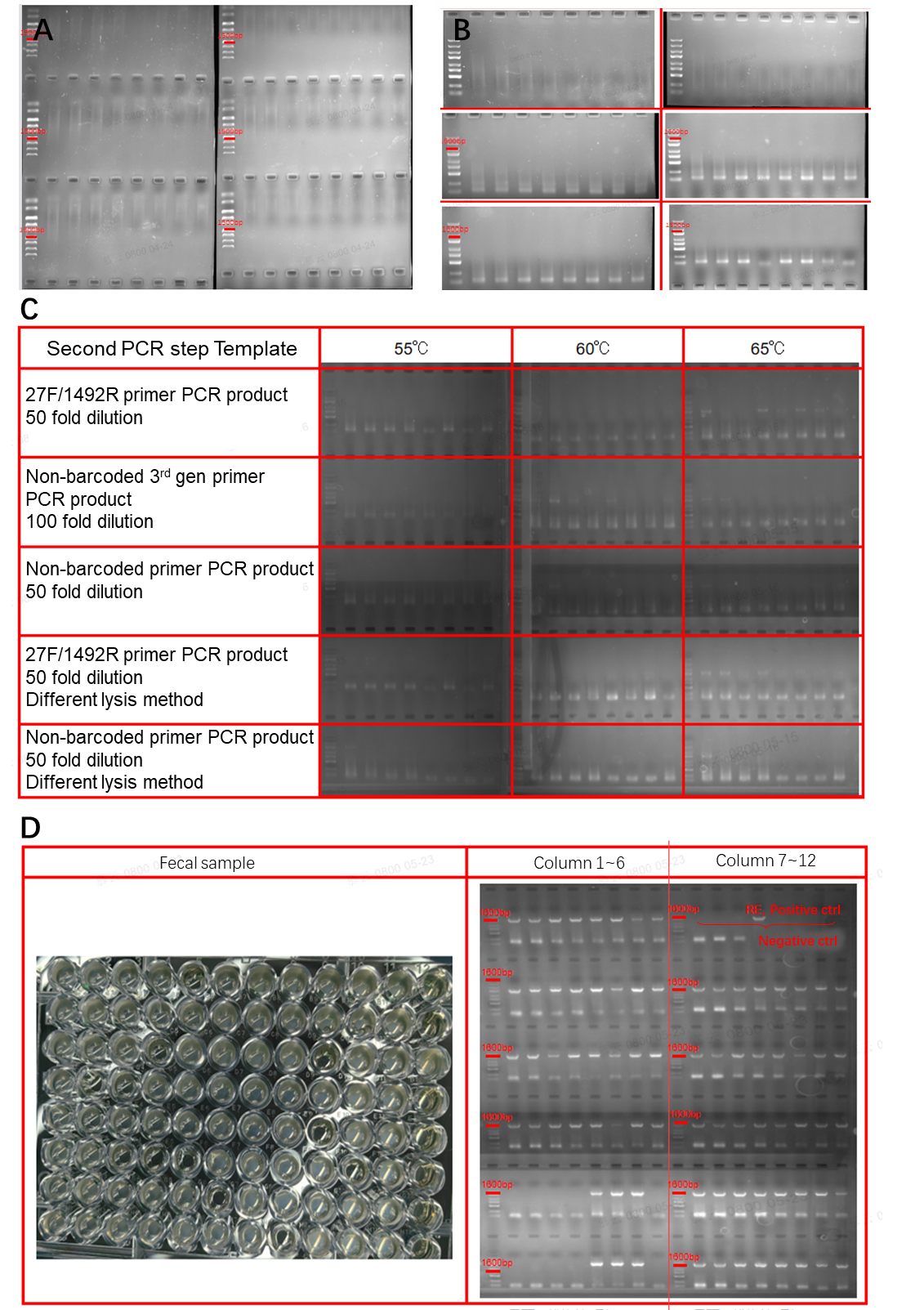


**Figure S1.** Optimization of high-throughput PCR workflow. (A) PCR amplification using the 27F/1492R primers. (B) Initial PCR amplification using double-ended barcoded primers before condition optimization. (C) Two-step PCR method condition optimization: bacterial cells were lysed, and the 27F/1492R or non-barcoded primers were used for the first round of amplification. The diluted amplification products were then used as templates for a second round of PCR with double-ended barcoded primers (Two-step method). (D) Final optimized PCR amplification using the double-ended barcoded primers in a single-step PCR method (One-step method).


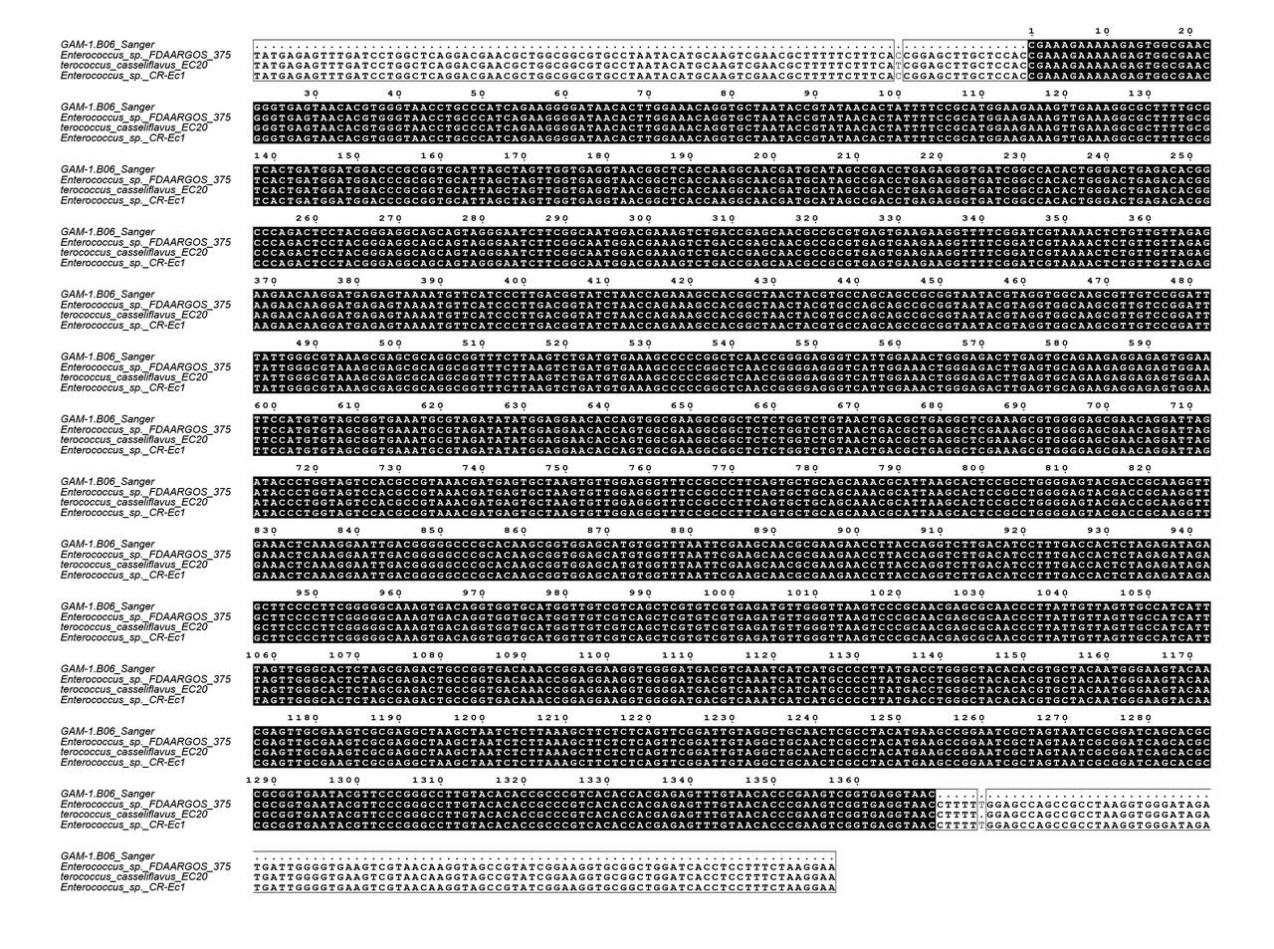


**Figure S2**. Multiple 16S rDNA sequence alignment from Sanger and species in database of *Emu*. These sequences were obtained from Sanger sequencing, and showed high similarity (>99%) to multiple species in the database.


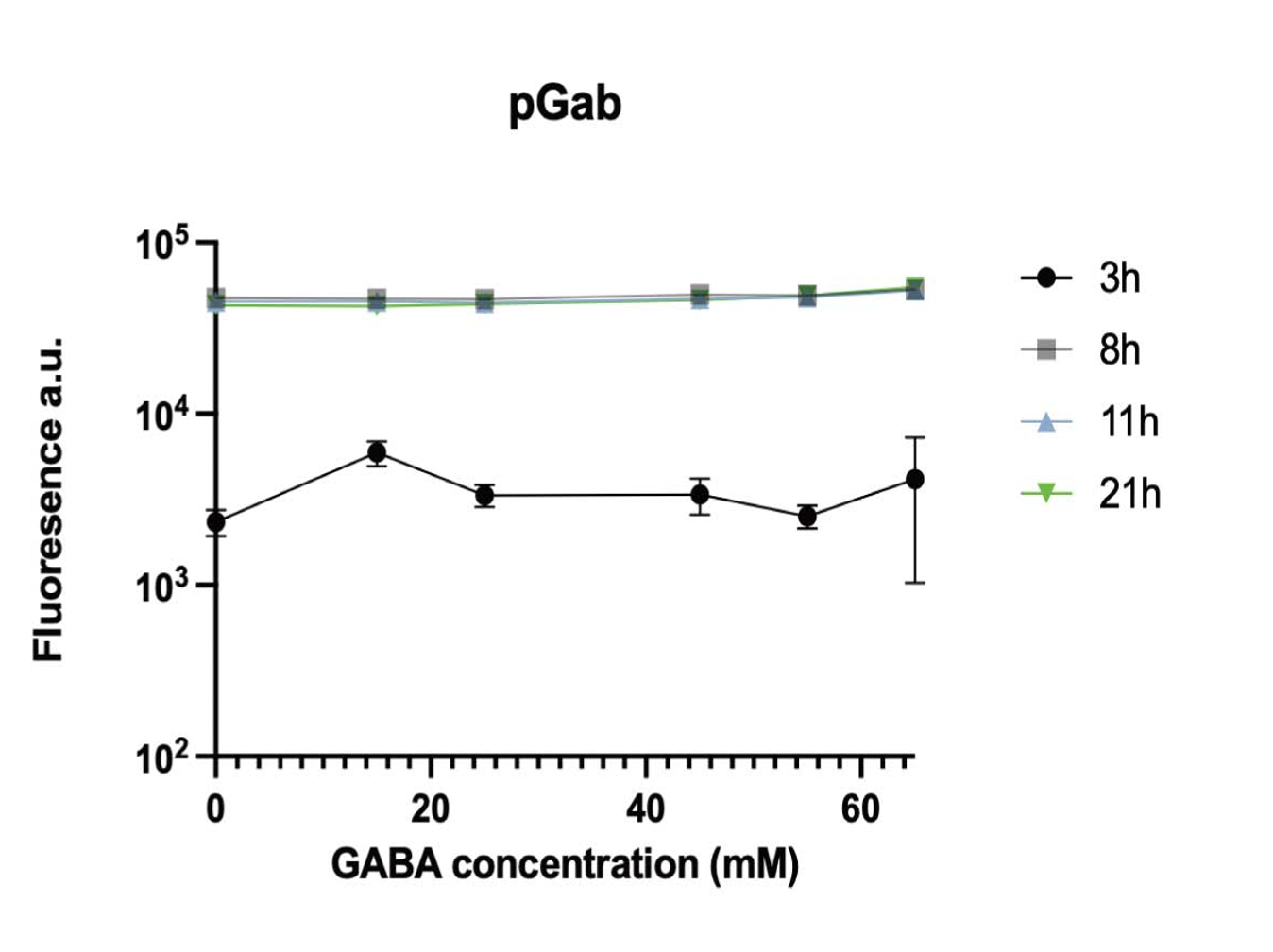


**Figure S3.** The sfGFP expression levels in response to different GABA concentrations, in the presence of the reporter plasmid and the absence of the sensor plasmid in *E. coli*. Lines denoted as 3h, 8h, 11h and 21h represent samples taken after 3 hours, 8 hours, 11 hours and 21 hours after the start of cultivation, respectively. The data were gathered using *E. coli* DH5α chassis in LB medium, with a fixed IPTG concentration of 0.005 mM added at the beginning of cultivation.

**Figure S4**. The sfGFP expression levels in response to different GABA concentrations with different IPTG concentrations, carried out using *E. coli* Nissle 1917 and cultured in M9 medium for 6 hours. The IPTG was added at the start of cultivation, n = 4.


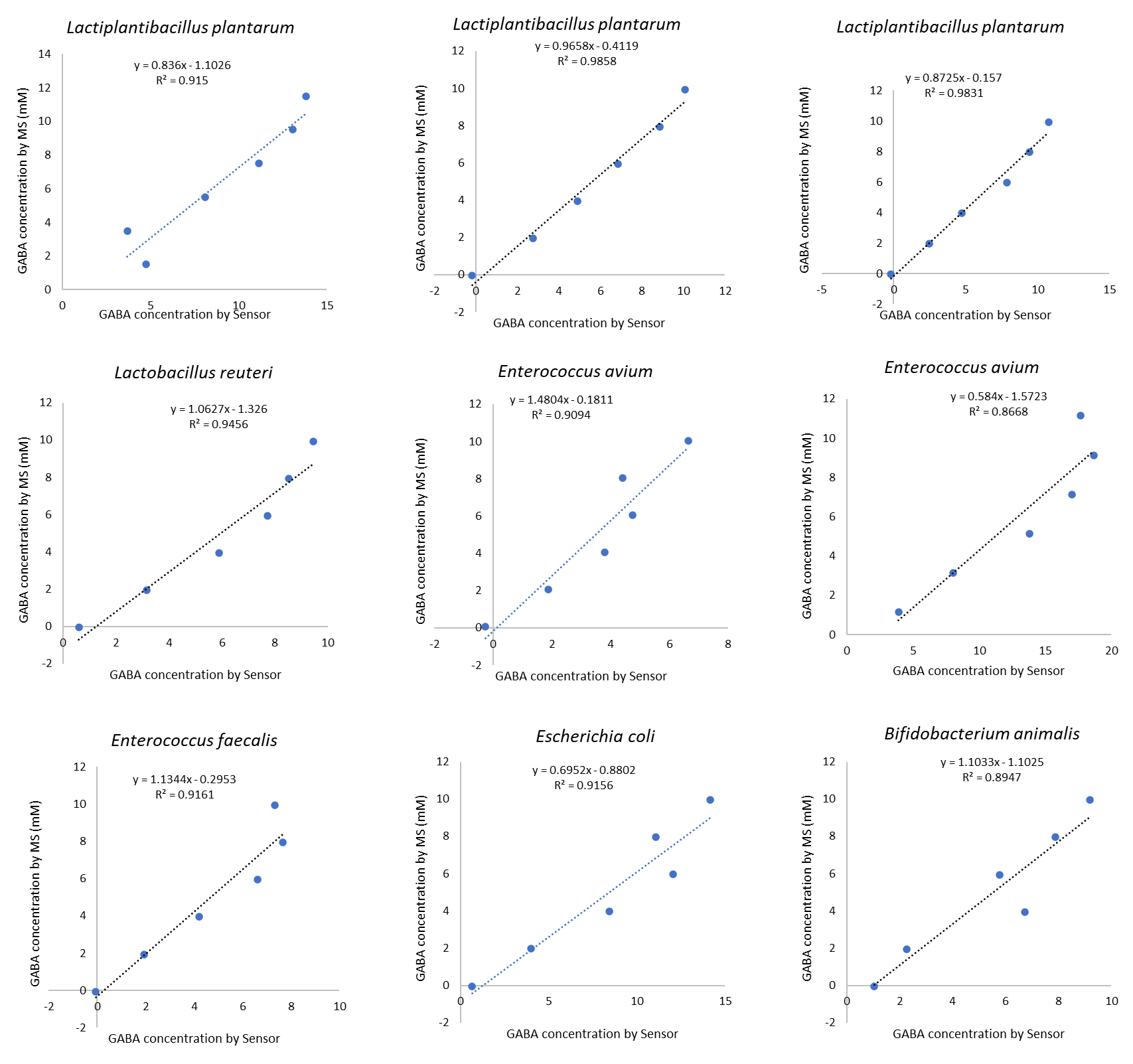


**Figure S5.** Linear regression of GABA concentrations detected by GABA biosensor compared to mass spectrometry. Sample was divided into two identical portions, one used for mass spectrum analysis and the other portion is used for biosensor cell cultivation. Biosensor measurements were taken after 6 hours of cultivation in MRS medium.


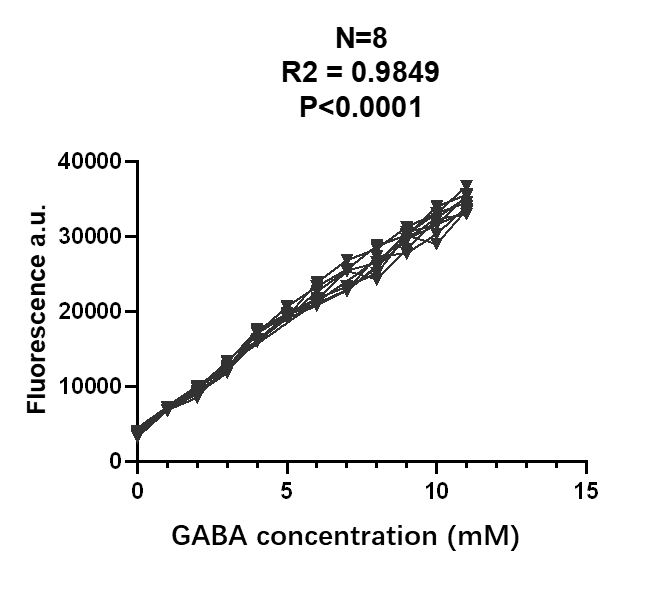


**Figure S6**. Calibration curve of mass spectrometry-determined GABA concentrations against GABA biosensor fluorescence readout. Known amount of GABA standard was spiked in BHI culture media and cultured with GABA biosensor for 6 hours, and then tested using both UPLC-HRMS and GABA biosensor.


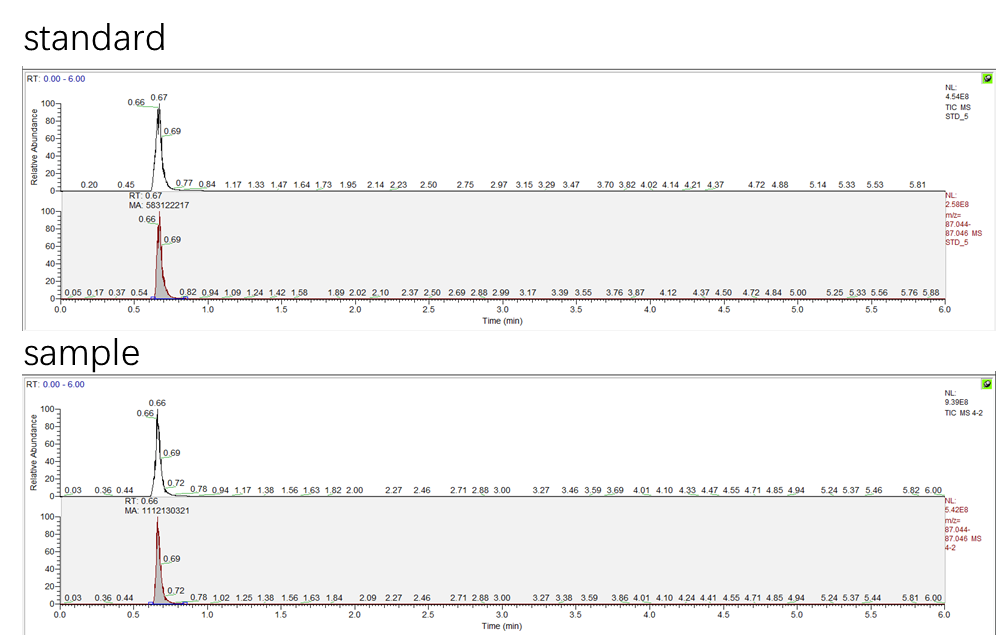


**Figure S7.** Representative MS EIC of GABA standard (0.5 g/L) and bacterial sample (B9-MH10-F1)


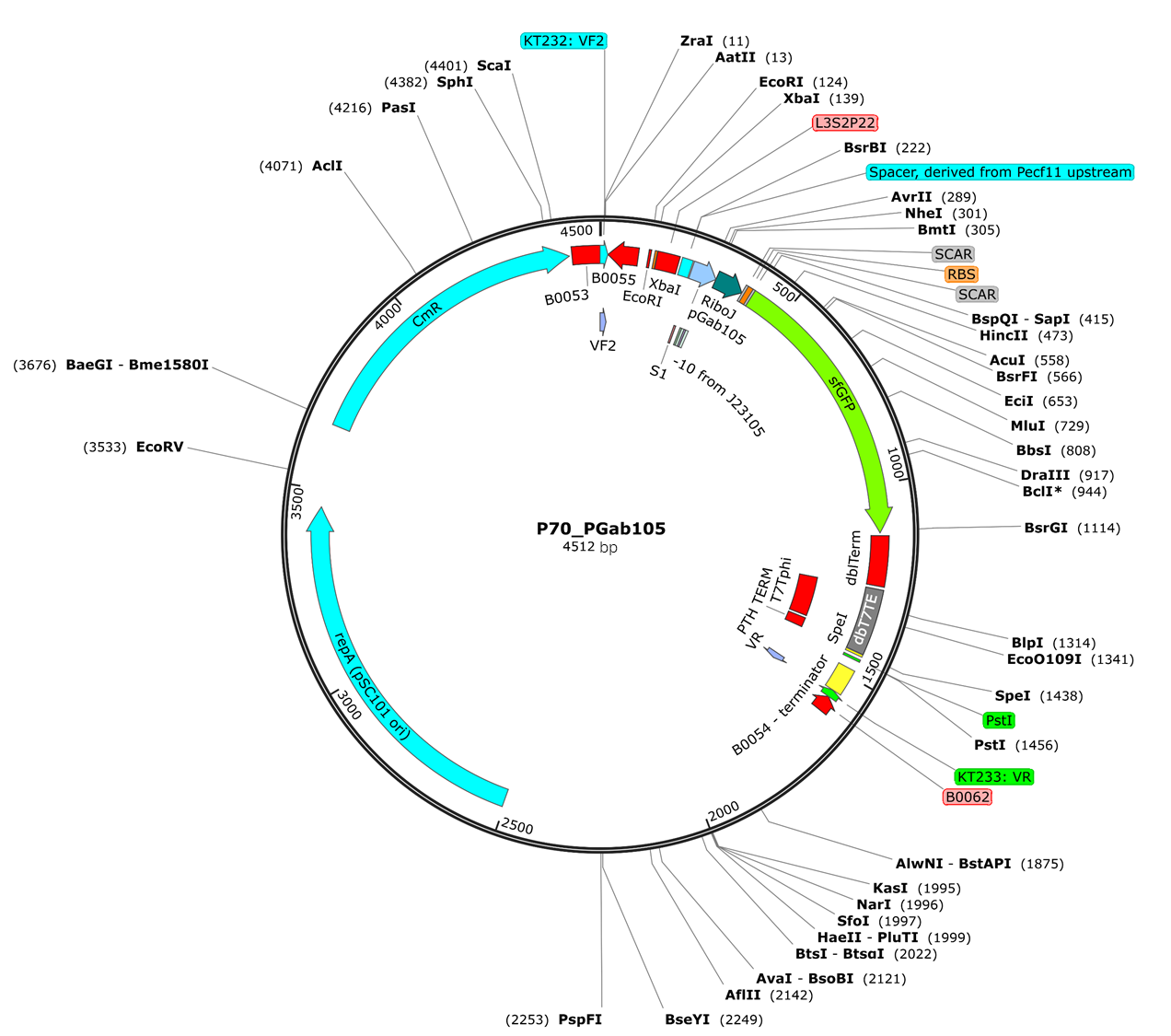


**Figure S8.** Plasmid map of the reporter plasmid


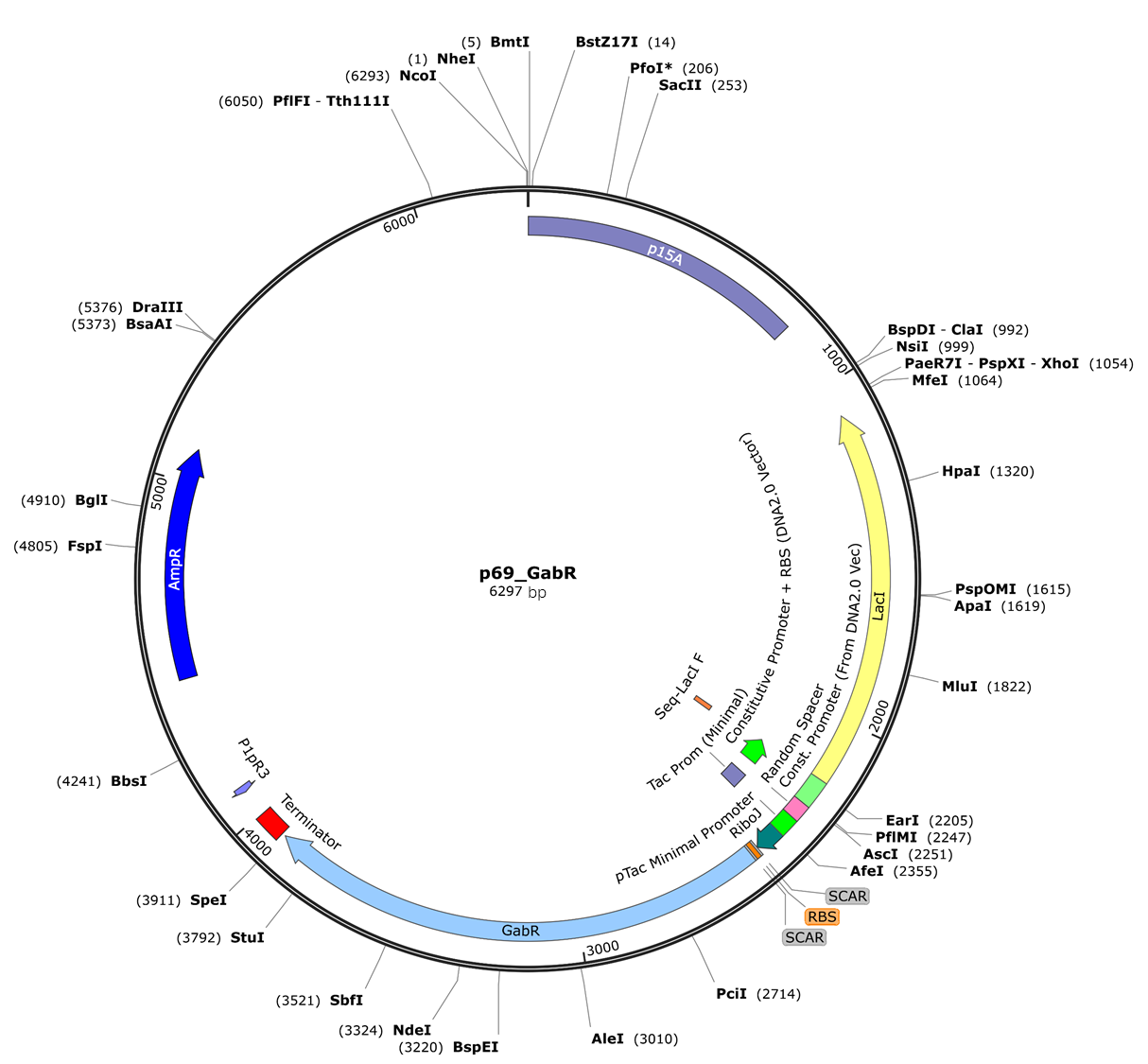


**Figure S9.** Plasmid map for the sensor plasmid.

**Table S1** Demultiplexing summary of Nanopore for large scale species identification of Biobank

| **Sample** | **pooled sample number** | **Total reads** | **proper length reads (>1400bp and <1800bp)** | **unique dual barcode reads** | **Multiple barcode reads** | **reads with only one barcode** | **reads without any barcode** | **Demultiplexing ratio** |
| --- | --- | --- | --- | --- | --- | --- | --- | --- |
| Nanopore MH1 | 2,304 | 29,583,692 | 20,725,980 | 37.65% | 0.25% | 47.44% | 14.66% | 28.00% |
| Nanopore MH2 | 2,304 | 54,752,620 | 14,056,868 | 41.85% | 1.32% | 45.07% | 11.76% | 36.06% |
| Nanopore MH3 | 2,304 | 34,758,131 | 21,428,878 | 38.08% | 0.50% | 46.82% | 14.60% | 31.35% |
| Nanopore MH4 | 2,304 | 34,465,945 | 19,310,099 | 32.63% | 0.35% | 49.05% | 17.97% | 24.94% |
| Nanopore MH5 | 2,304 | 42,223,428 | 16,984,552 | 32.44% | 0.19% | 49.94% | 17.43% | 25.87% |
| Nanopore MH6 | 2,304 | 32,716,631 | 19,307,634 | 37.44% | 0.30% | 47.37% | 14.90% | 30.47% |
| Nanopore MH7 | 2,304 | 37,060,860 | 15,115,780 | 33.87% | 0.46% | 49.55% | 16.13% | 27.75% |
| Nanopore MH8 | 2,304 | 27,884,895 | 21,807,483 | 43.55% | 0.25% | 43.92% | 12.28% | 36.06% |
| Nanopore MH9 | 2,304 | 28,822,512 | 20,913,244 | 41.08% | 0.15% | 45.38% | 13.39% | 33.50% |
| Nanopore MH10 | 2,304 | 27,796,545 | 21,422,908 | 38.53% | 0.17% | 46.84% | 14.46% | 30.93% |
